# Supplementary material for: Radiosynthesis and Bioevaluation of 99mTc-Labeled Isocyanide Ubiquicidin 29-41 Derivatives as Potential Agents for Bacterial Infection Imaging
Source: Int J Mol Sci. 2024 Jan 15;25(2):1045. doi: 10.3390/ijms25021045 (PMC10816394; doi:10.3390/ijms25021045)
Supplement: Supplementary file 1 [file ijms-25-01045-s001.zip › ijms-2805725-supplementary.pdf]

Radiosynthesis and Bioevaluation of ^{99m}Tc-Labeled Isocyanide Ubiquicidin 29-41 Derivatives as Potential Agents for Bacterial Infection Imaging

Yuhao Jiang [1,†], Peiwen Han [1,†], Guangxing Yin [1], Qianna Wang [1], Junhong Feng [1,2], Qing Ruan [1,3], Di Xiao [1] and Junbo Zhang [1,*]

[1] Key Laboratory of Radiopharmaceuticals of Ministry of Education, NMPA Key Laboratory for Research and Evaluation of Radiopharmaceuticals (National Medical Products Administration), College of Chemistry, Beijing Normal University, Beijing 100875, China; 202131150056@mail.bnu.edu.cn (Y.J.); 202221150118@mail.bnu.edu.cn (P.H.); 202331150067@mail.bnu.edu.cn (G.Y.); 202231150061@mail.bnu.edu.cn (Q.W.); 201921150102@mail.bnu.edu.cn (J.F.); 11132022029@bnu.edu.cn (Q.R.); 202031150054@mail.bnu.edu.cn (D.X.)

[2] Department of Isotopes, China Institute of Atomic Energy, P.O. Box 2108, Beijing 102413, China

[3] Key Laboratory of Beam Technology of the Ministry of Education, College of Nuclear Science and Technology, Beijing Normal University, Beijing 100875, China

* Correspondence: zhjunbo@bnu.edu.cn; Tel.: +86-10-6220-8126

Content

Figure S1. ESI-MS spectrum of compound L1 CN5UBI 29-41 2

Figure S2. UV-HPLC chromatogram of compound L1 CN5UBI 29-41 3

Figure S3. ESI-MS spectrum of compound L2 CN6UBI 29-41 3

Figure S4. UV-HPLC chromatogram of compound L2 CN6UBI 29-41 4

Figure S5. ESI-MS spectrum of compound L3 CN7UBI 29-41 4

Figure S6. UV-HPLC chromatogram of compound L3 CN7UBI 29-41 5

Figure S7. ESI-MS spectrum of compound L4 CN8UBI 29-41 5

Figure S8. UV-HPLC chromatogram of compound L4 CN8UBI 29-41 6

Figure S9. ESI-MS spectrum of compound L5 CN9UBI 29-41 6

Figure S10. UV-HPLC chromatogram of compound L5 CN9UBI 29-41 7

Figure S11. Radio-HPLC chromatograms of [^{99m}Tc][TcO_4]$^-$ and [^{99m}Tc][TcN]$^{2+}$ in HPLC system 1 7

Figure S12. Radio-HPLC chromatograms of [^{99m}Tc][TcO_4]$^-$ and [^{99m}Tc][$Tc(CO)_3(H_2O)_3$]$^+$ in HPLC system 2 7

Figure S13. Radio-HPLC chromatograms of [^{99m}Tc]Tc-CNnUBI 29-41 8

Figure S14. Radio-HPLC chromatograms of [^{99m}Tc]$Tc(CO)_3$-CNnUBI 29-41 9

Figure S15. Radio-HPLC chromatograms of [^{99m}Tc]TcN-CNnUBI 29-41 10

Table S1. The retention time of the ^{99m}Tc-labeled complexes 10

Figure S16. *In vitro* stability in saline and mouse serum 6 h HPLC chromatograms of [99mTc]Tc-CNnUBI 29-41 ....**11**

Figure S17. *In vitro* stability in saline and mouse serum 4 h HPLC chromatograms of [99mTc]Tc(CO)3-CNnUBI 29-41..........**11**

Figure S18. *In vitro* stability in saline and mouse serum 4 h HPLC chromatograms of [99mTc]TcN-CNnUBI 29-41 ....**12**

Table S2. Biodistribution results of [99mTc]Tc-CNnUBI 29-41 in *S. aureus*-infected mice ...**13**

Table S3. Biodistribution results of [99mTc]Tc(CO)3-CNnUBI 29-41 in *S. aureus*-infected mice..........**14**

Table S4. Biodistribution results of [99mTc]TcN-CNnUBI 29-41 in *S. aureus*-infected mice **15**

Table S5. Biodistribution results of [99mTc]Tc-CN5UBI 29-41 in *S. aureus*-infected and turpentine-oil-inflamed mice **16**

Figure S19. SPECT image of [99mTc]Tc(CO)3-CN5UBI 29-41 in mice with bacterial infection at 120 min postinjection **17**

Figure S20. SPECT image of [99mTc]TcN-CN5UBI 29-41 in mice with bacterial infection at 120 min postinjection..........**18**

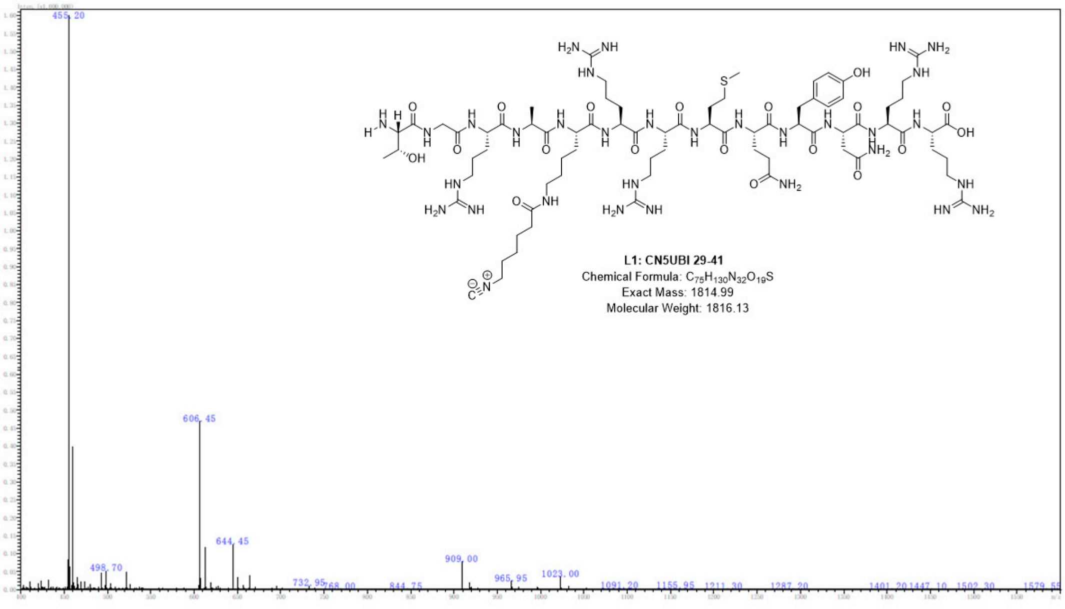

Figure S1. ESI-MS spectrum of compound L1 CN5UBI 29-41

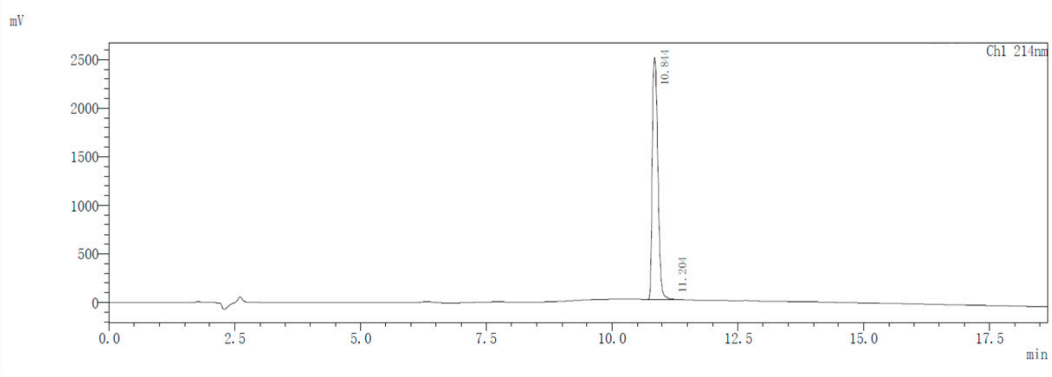

Figure S2. UV-HPLC chromatogram of compound L1 CN5UBI 29-41

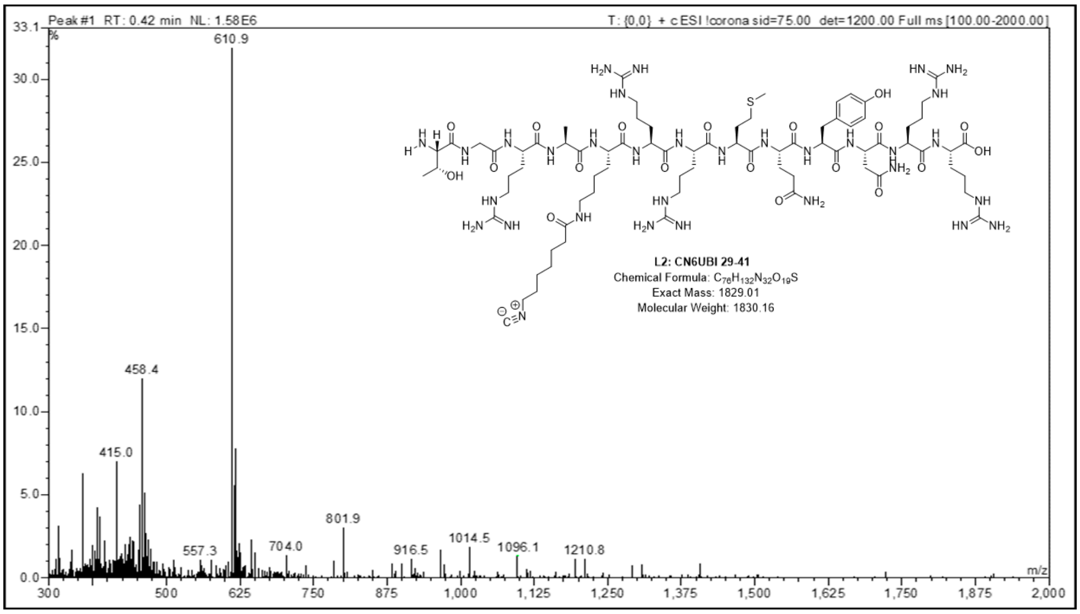

Figure S3. ESI-MS spectrum of compound L2 CN6UBI 29-41

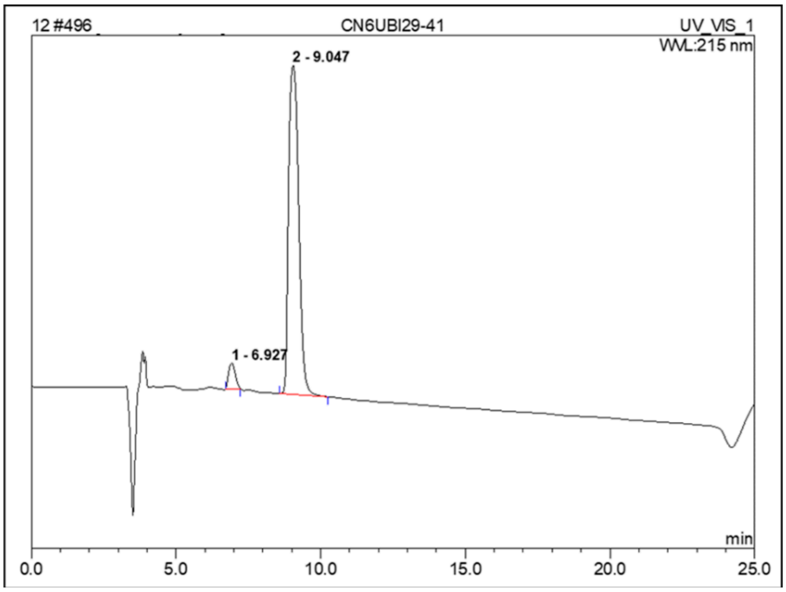

Figure S4. UV-HPLC chromatogram of compound L2 CN6UBI 29-41

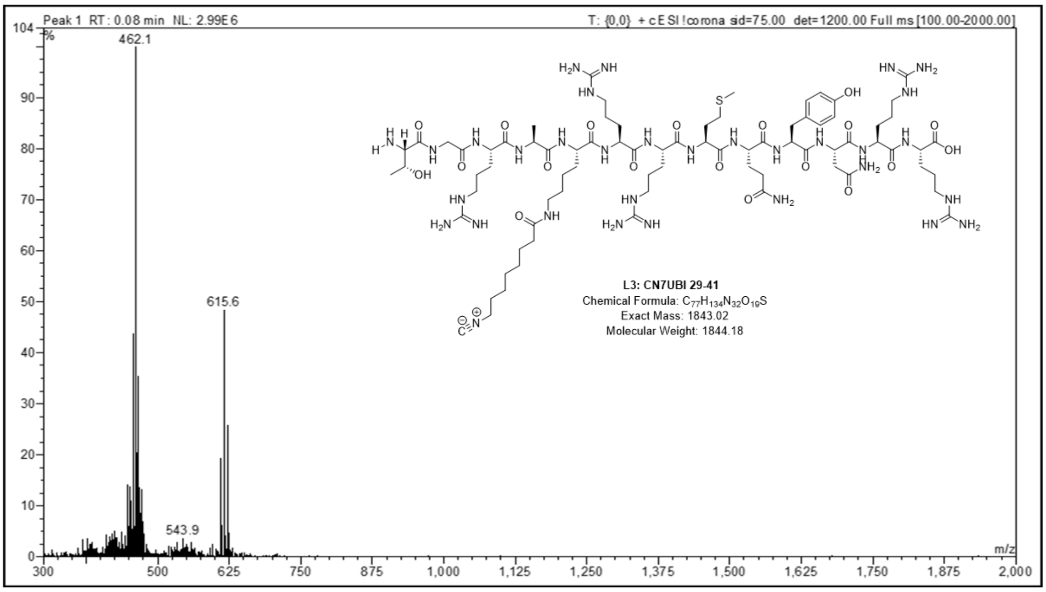

Figure S5. ESI-MS spectrum of compound L3 CN7UBI 29-41

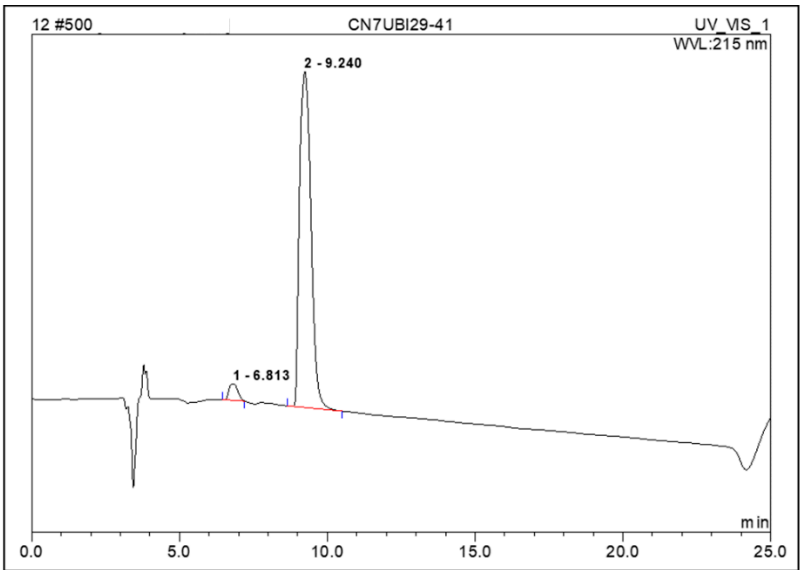

Figure S6. UV-HPLC chromatogram of compound L3 CN7UBI 29-41

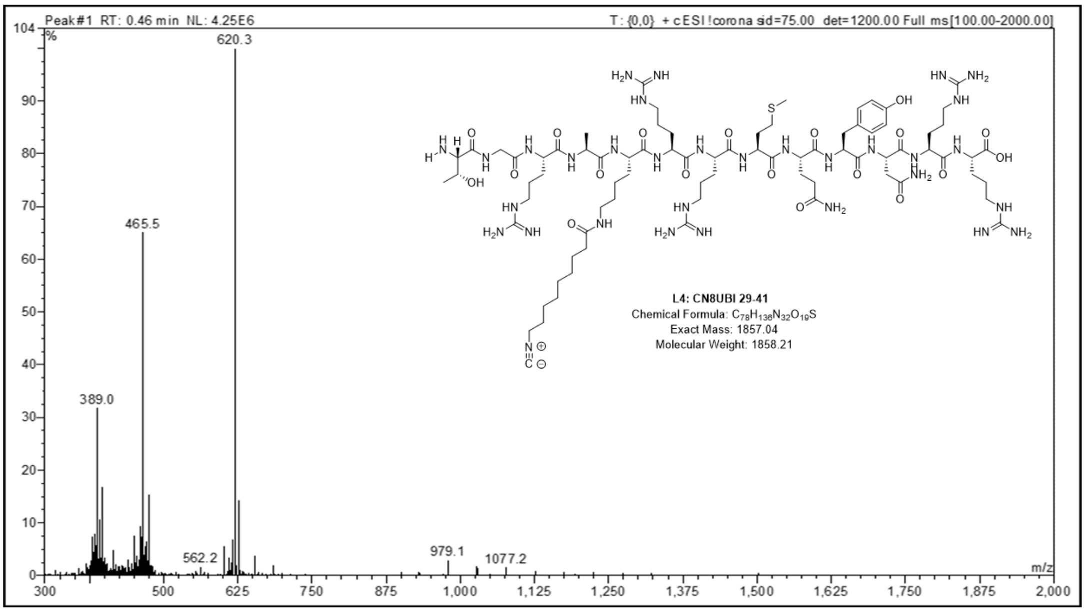

Figure S7. ESI-MS spectrum of compound L4 CN8UBI 29-41

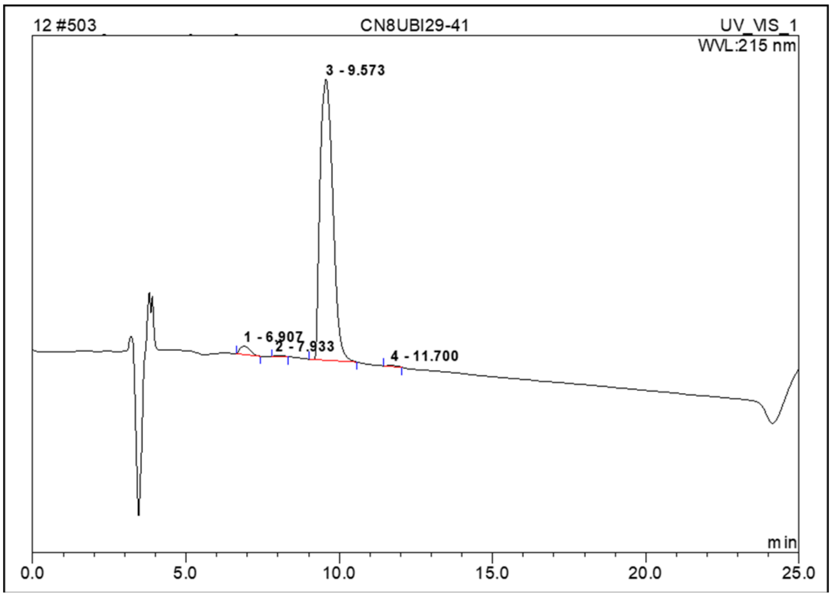

Figure S8. UV-HPLC chromatogram of compound L4 CN8UBI 29-41

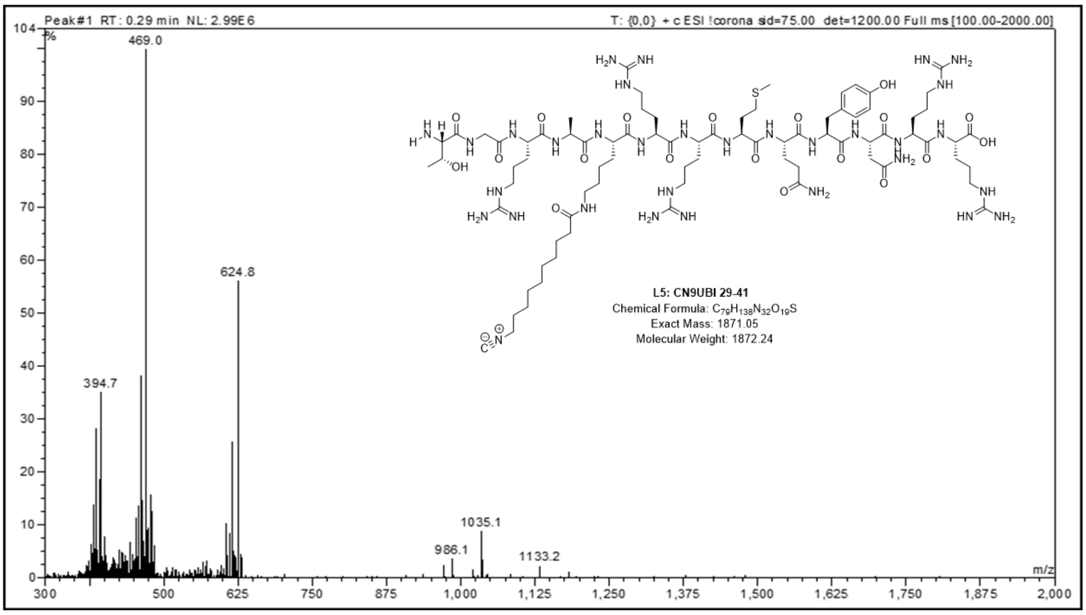

Figure S9. ESI-MS spectrum of compound L5 CN9UBI 29-41

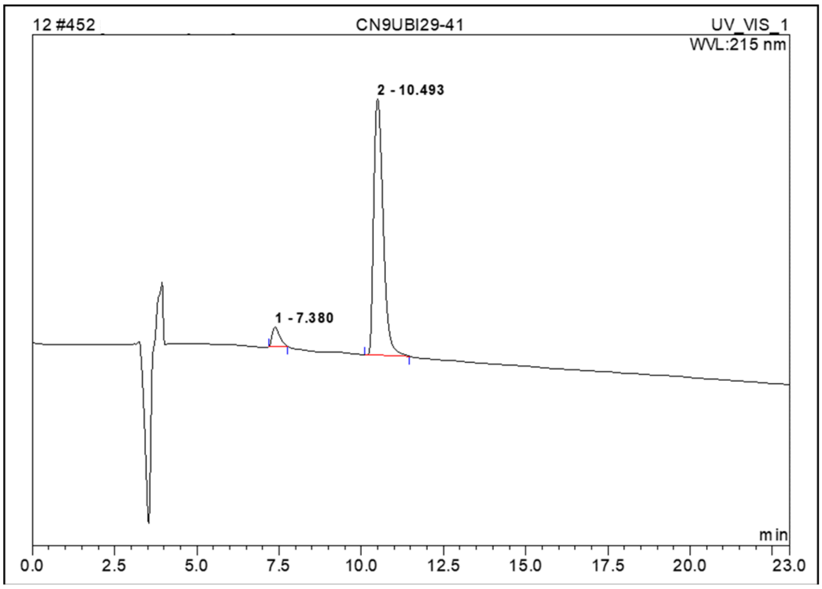

Figure S10. UV-HPLC chromatogram of compound L5 CN9UBI 29-41

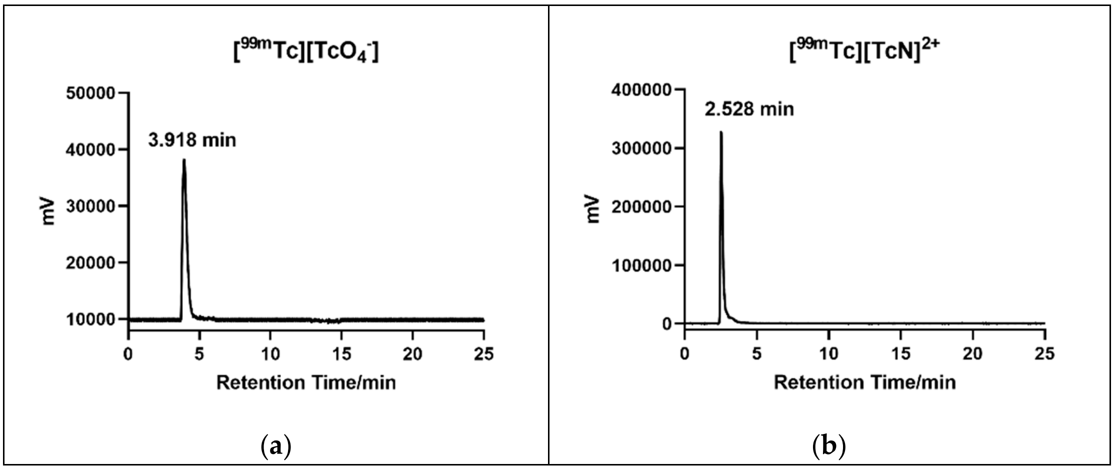

Figure S11. Radio-HPLC chromatograms of [^{99m}Tc]TcO_4^- (**a**) and [^{99m}Tc][TcN]$^{2+}$ (**b**) in HPLC system 1.

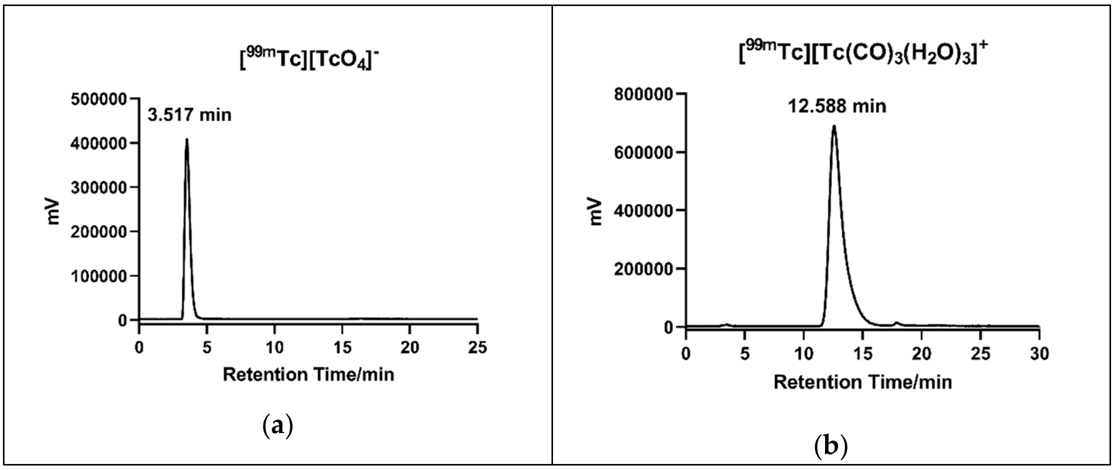

Figure S12. Radio-HPLC chromatograms of [^{99m}Tc]TcO_4^- (**a**) and [^{99m}Tc][$Tc(CO)_3(H_2O)_3$]$^+$ (**b**) in HPLC system 2.

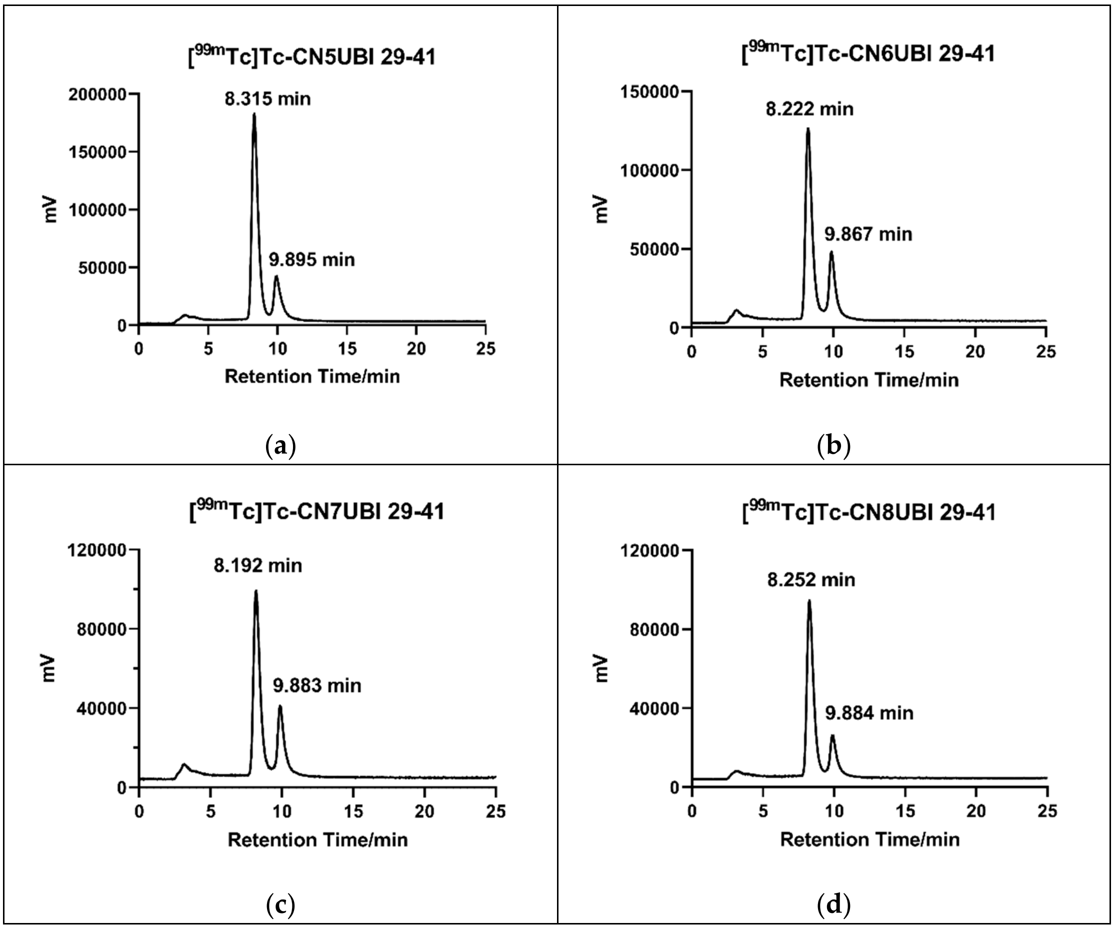

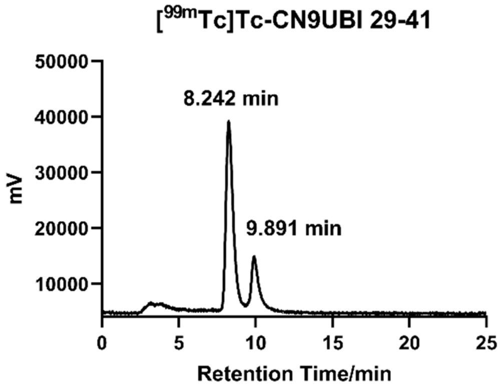

(**e**)

Figure S13. Radio-HPLC chromatograms of [^{99m}Tc]Tc-CNnUBI 29-41 (n = 5-9).

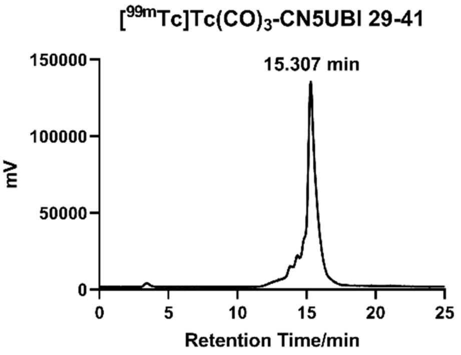

(**a**)

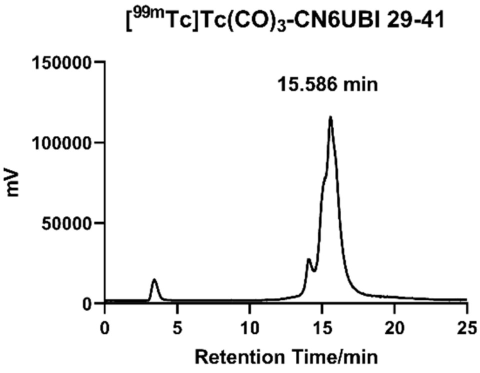

(**b**)

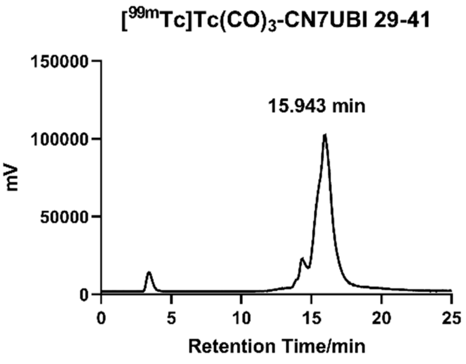

(**c**)

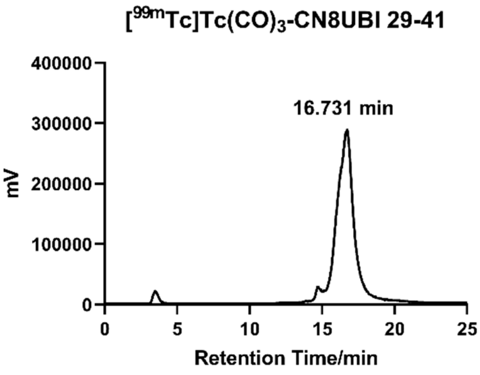

(**d**)

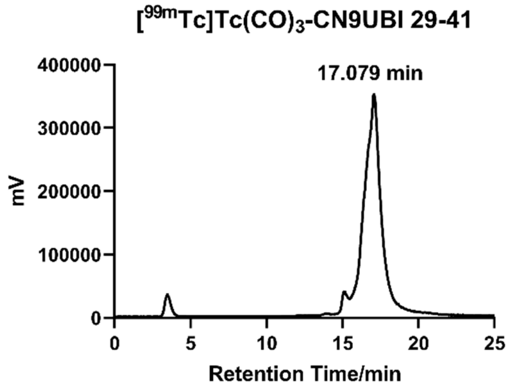

(**e**)

Figure S14. Radio-HPLC chromatograms of [^{99m}Tc]Tc(CO)$_3$-CNnUBI 29-41 (*n* = 5-9).

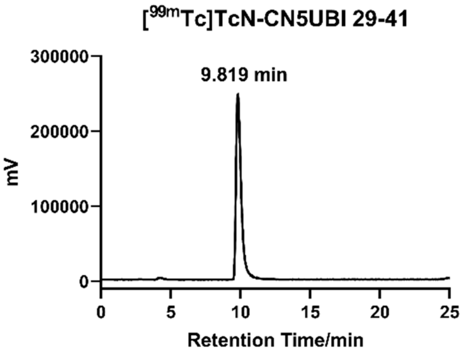

(**a**)

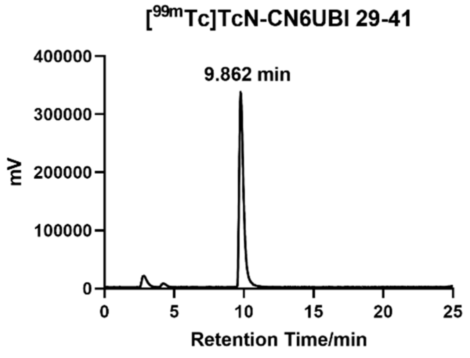

(**b**)

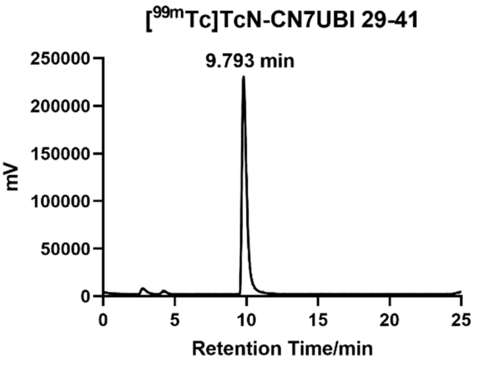

(**c**)

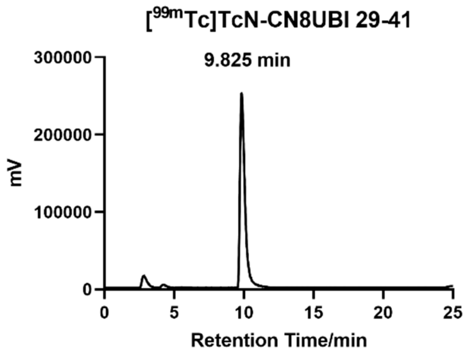

(**d**)

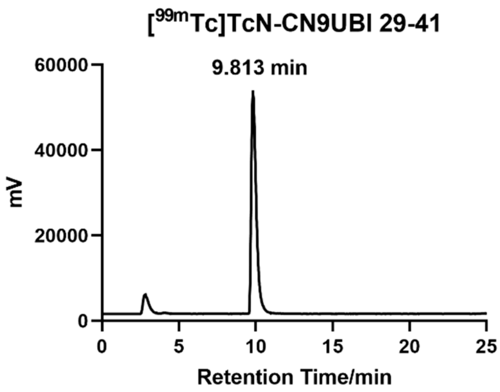

(e)

Figure S15. Radio-HPLC chromatograms of [^{99m}Tc]TcN-CNnUBI 29-41 (n = 5-9).

Table S1. The retention time of the ^{99m}Tc-labeled complexes.

Ligand	[^{99m}Tc][Tc(I)]$^+$ core	[^{99m}Tc][Tc(CO)$_3$(H$_2$O)$_3$]$^+$ core	[^{99m}Tc][Tc(V)N]$^{2+}$ core
L1: CN5UBI 29-41	8.315 min, 9.895 min	15.307 min	9.819 min
L2: CN6UBI 29-41	8.222 min, 9.867 min	15.586 min	9.862 min
L3: CN7UBI 29-41	8.192 min, 9.883 min	15.943 min	9.793 min
L4: CN8UBI 29-41	8.252 min, 9.884 min	16.731 min	9.825 min
L5: CN9UBI 29-41	8.242 min, 9.891 min	17.039 min	9.813 min

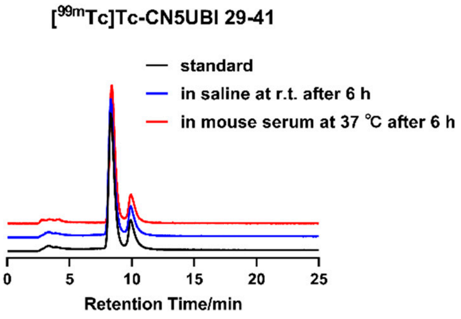

(a)

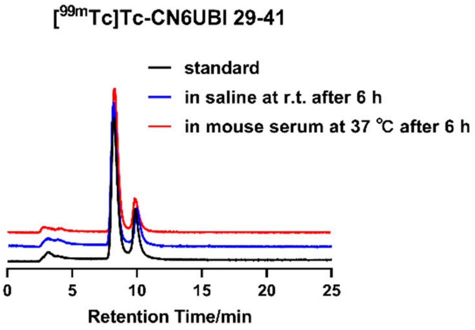

(b)

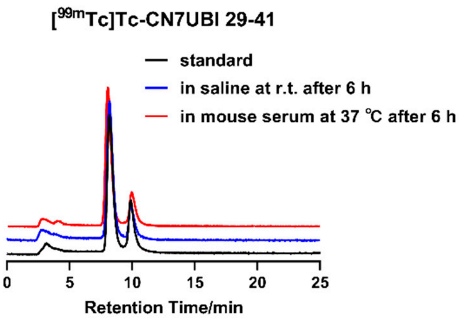

(c)

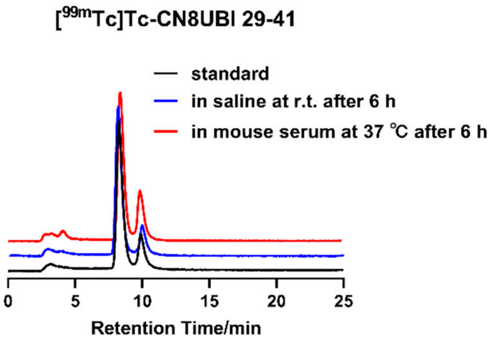

(d)

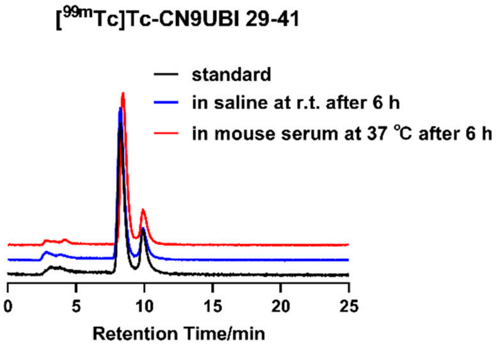

(**e**)

Figure S16. *In vitro* stability in saline and mouse serum 6 h HPLC chromatograms of [^{99m}Tc]Tc-CNnUBI 29-41 (*n* = 5-9).

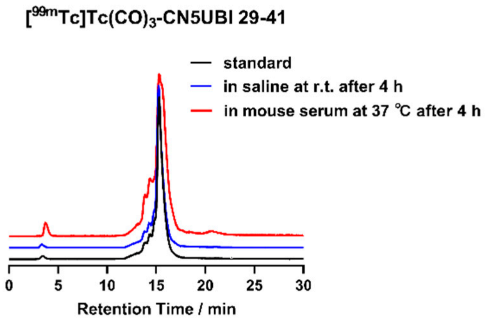

(**a**)

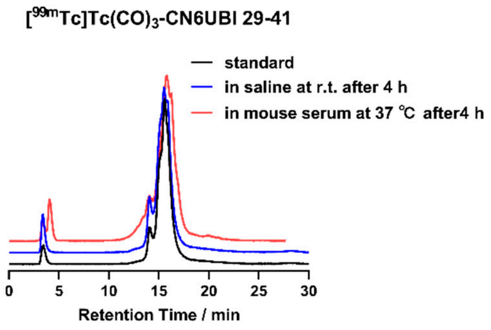

(**b**)

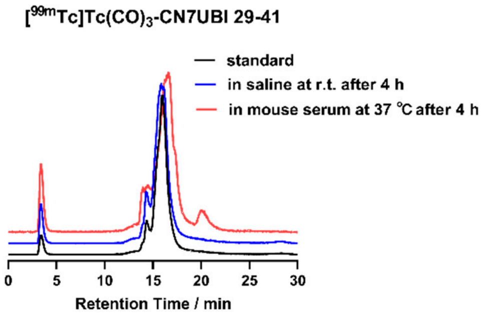

(**c**)

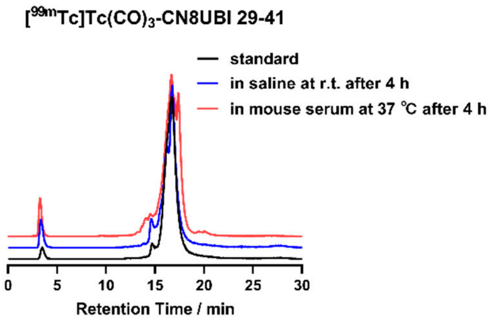

(**d**)

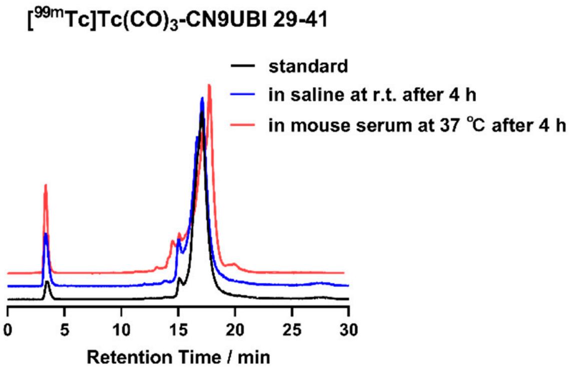

(**e**)

Figure S17. *In vitro* stability in saline and mouse serum 4 h HPLC chromatograms of [^{99m}Tc]Tc(CO)$_3$-CNnUBI 29-41 (*n* = 5-9).

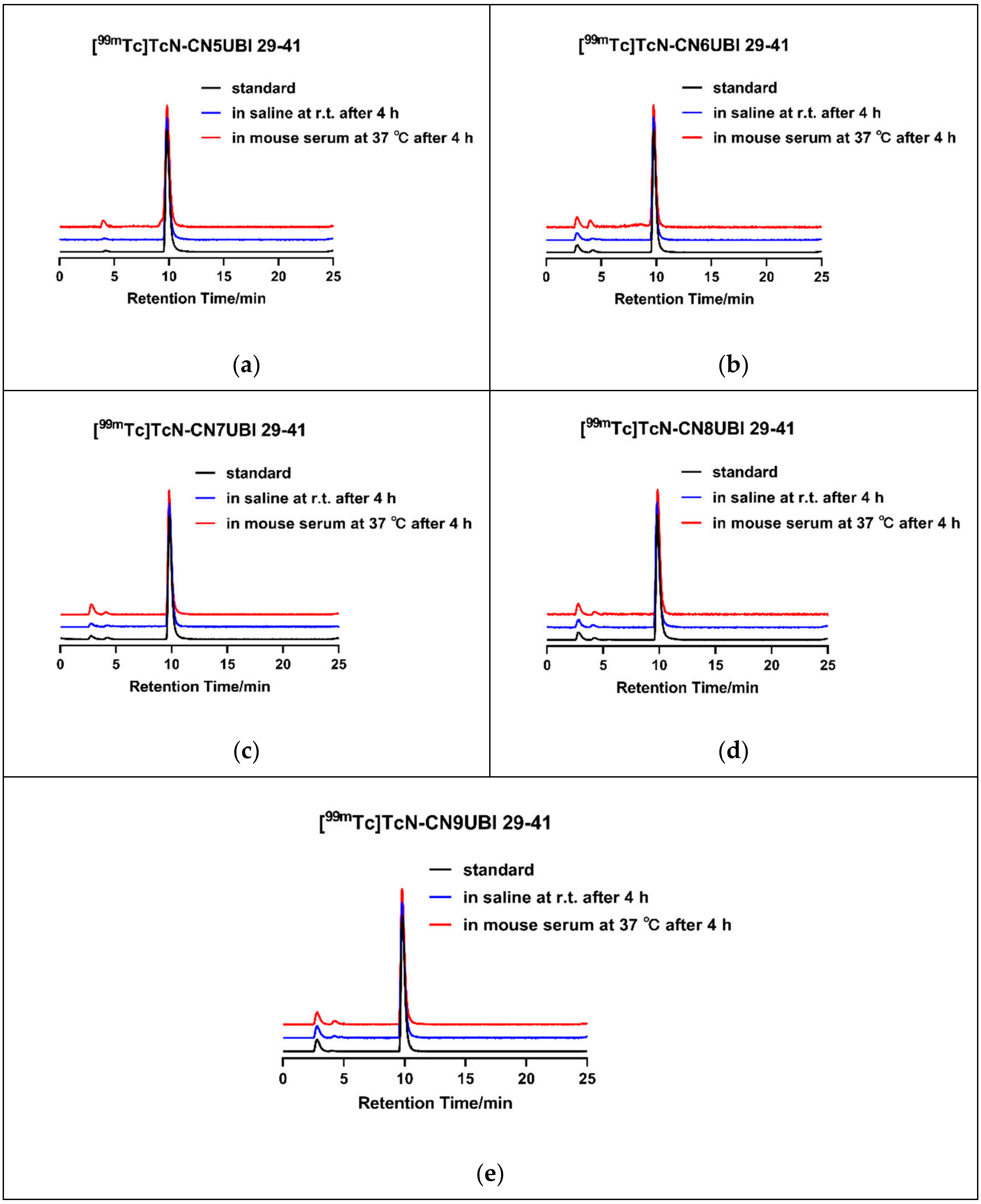

Figure S18. *In vitro* stability in saline and mouse serum 4 h HPLC chromatograms of [^{99m}Tc]TcN-CNnUBI 29-41 (*n* = 5-9).

Table S2. Biodistribution results of [^{99m}Tc]Tc-CNnUBI 29-41 in *S. aureus*-infected mice (mean ± SD, %ID/g, n = 5, 120 min p.i.)

Tissues	n = 5	n = 6	n = 7	n = 8	n = 9
Heart	0.89 ± 0.25	0.97 ± 0.09	0.51 ± 0.04	0.64 ± 0.02	0.77 ± 0.13
Liver	2.24 ± 0.67	6.92 ± 0.94	5.57 ± 1.00	7.36 ± 0.78	6.25 ± 0.93
Lung	1.85 ± 0.29	2.05 ± 0.15	1.47 ± 0.27	2.00 ± 0.51	2.32 ± 0.66
Kidney	18.00 ± 3.49	44.78 ± 3.59	28.29 ± 3.41	30.51 ± 2.24	29.10 ± 2.91
Spleen	0.77 ± 0.19	2.98 ± 0.42	2.28 ± 0.65	2.78 ± 0.91	2.90 ± 0.61
Stomach	0.70 ± 0.24	0.64 ± 0.16	0.56 ± 0.13	0.97 ± 0.28	0.84 ± 0.43
Bone	1.06 ± 0.14	1.30 ± 0.07	0.93 ± 0.13	1.01 ± 0.07	1.01 ± 0.16
Muscle	0.34 ± 0.03	0.50 ± 0.06	0.34 ± 0.06	0.39 ± 0.04	0.39 ± 0.09
Small Intestines	1.65 ± 0.95	2.02 ± 0.49	1.97 ± 0.68	3.03 ± 0.57	2.76 ± 1.16
Large Intestines	2.23 ± 1.02	4.23 ± 0.93	4.08 ± 1.09	3.14 ± 0.71	4.96 ± 1.33
Abscess	1.44 ± 0.46	1.70 ± 0.23	0.81 ± 0.18	0.99 ± 0.24	1.08 ± 0.23
Blood	1.81 ± 0.29	2.19 ± 0.11	1.35 ± 0.15	1.42 ± 0.09	1.68 ± 0.32
Gallbladder (%ID)	0.13 ± 0.10	0.03 ± 0.01	0.10 ± 0.18	0.58 ± 0.18	0.55 ± 0.36
Thyroid (%ID)	0.02 ± 0.01	0.02 ± 0.01	0.01 ± 0.00	0.02 ± 0.00	0.02 ± 0.00
Abscess/Muscle	4.15 ± 0.49	3.48 ± 0.70	2.52 ± 0.56	2.64 ± 0.65	2.62 ± 0.42
Abscess/Blood	0.81 ± 0.05	0.75 ± 0.10	0.56 ± 0.09	0.66 ± 0.16	0.69 ± 0.04

Table S3. Biodistribution results of [^{99m}Tc]Tc(CO)$_3$-CNnUBI 29-41 in *S. aureus*-infected mice (mean ± SD, %ID/g, n = 5, 120 min p.i.)

Tissues	n = 5	n = 6	n = 7	n = 8	n = 9
Heart	2.04 ± 0.23	1.25 ± 0.14	1.45 ± 0.17	1.36 ± 0.04	2.04 ± 0.17

Tissues	n = 5	n = 6	n = 7	n = 8	n = 9
Liver	5.81 ± 0.44	10.83 ± 1.96	14.57 ± 4.57	14.37 ± 1.49	18.83 ± 1.56
Lung	5.03 ± 0.97	3.82 ± 0.68	5.47 ± 1.08	3.65 ± 0.25	5.97 ± 1.17
Kidney	16.35 ± 1.46	38.86 ± 4.50	35.63 ± 4.16	25.07 ± 0.99	24.83 ± 1.90
Spleen	2.42 ± 0.23	5.71 ± 1.29	5.91 ± 0.76	5.39 ± 1.25	8.69 ± 1.35
Stomach	2.84 ± 0.68	2.76 ± 0.43	3.32 ± 1.33	2.56 ± 1.13	6.83 ± 2.63
Bone	2.38 ± 0.42	1.36 ± 0.68	2.15 ± 0.26	1.79 ± 0.22	2.58 ± 0.22
Muscle	1.44 ± 0.51	0.69 ± 0.10	0.80 ± 0.21	0.57 ± 0.06	0.97 ± 0.25
Small Intestines	3.19 ± 0.78	6.31 ± 1.17	5.11 ± 2.07	4.39 ± 1.87	5.69 ± 1.79
Large Intestines	3.28 ± 1.24	3.54 ± 1.17	1.83 ± 0.67	1.84 ± 1.00	3.28 ± 1.91
Abscess	3.44 ± 0.89	1.70 ± 0.34	1.86 ± 0.16	1.33 ± 0.24	2.41 ± 0.26
Blood	5.75 ± 0.98	2.36 ± 0.37	2.70 ± 0.27	2.07 ± 0.06	3.25 ± 0.26
Gallbladder (%ID)	0.90 ± 0.34	0.37 ± 0.27	0.67 ± 0.44	0.39 ± 0.16	1.65 ± 0.88
Thyroid (%ID)	0.15 ± 0.06	0.10 ± 0.03	0.09 ± 0.05	0.10 ± 0.02	0.22 ± 0.07
Abscess/Muscle	2.49 ± 0.65	2.48 ± 0.45	2.43 ± 0.49	2.42 ± 0.37	2.60 ± 0.68
Abscess/Blood	0.67 ± 0.15	0.73 ± 0.19	0.69 ± 0.08	0.64 ± 0.11	0.75 ± 0.12

Table S4. Biodistribution results of [^{99m}Tc]TcN-CNnUBI 29-41 in *S. aureus*-infected mice (mean ± SD, %ID/g, n = 5, 120 min p.i.)

Tissues	n = 5	n = 6	n = 7	n = 8	n = 9
Heart	0.21 ± 0.02	0.22 ± 0.04	0.23 ± 0.01	0.18 ± 0.01	0.28 ± 0.03
Liver	2.63 ± 0.37	1.30 ± 0.28	1.75 ± 0.32	1.78 ± 0.14	3.26 ± 0.34

Tissues	n = 5	n = 6	n = 7	n = 8	n = 9
Lung	0.60 ± 0.06	0.70 ± 0.19	0.68 ± 0.05	0.75 ± 0.05	1.38 ± 0.16
Kidney	17.99 ± 1.21	13.30 ± 2.00	10.60 ± 1.61	7.44 ± 0.77	8.08 ± 0.89
Spleen	0.90 ± 0.21	0.49 ± 0.07	0.45 ± 0.10	0.84 ± 0.23	1.23 ± 0.38
Stomach	0.32 ± 0.23	0.54 ± 0.12	0.68 ± 0.22	0.39 ± 0.11	0.85 ± 0.21
Bone	0.41 ± 0.07	0.26 ± 0.04	0.33 ± 0.07	0.27 ± 0.04	0.37 ± 0.08
Muscle	0.09 ± 0.01	0.10 ± 0.02	0.11 ± 0.01	0.11 ± 0.06	0.12 ± 0.02
Small Intestines	1.10 ± 0.31	1.03 ± 0.37	1.38 ± 0.19	1.13 ± 0.81	2.46 ± 0.79
Large Intestines	0.96 ± 0.61	0.25 ± 0.15	0.59 ± 0.32	1.81 ± 1.24	1.36 ± 1.03
Abscess	0.18 ± 0.05	0.33 ± 0.12	0.28 ± 0.05	0.25 ± 0.06	0.32 ± 0.11
Blood	0.27 ± 0.02	0.47 ± 0.11	0.47 ± 0.05	0.34 ± 0.04	0.45 ± 0.06
Gallbladder (%ID)	0.19 ± 0.10	0.10 ± 0.03	0.15 ± 0.10	0.29 ± 0.15	0.20 ± 0.16
Thyroid (%ID)	0.01 ± 0.00	0.01 ± 0.00	0.01 ± 0.00	0.01 ± 0.00	0.01 ± 0.00
Abscess/Muscle	2.10 ± 0.80	3.32 ± 0.99	2.47 ± 0.47	2.81 ± 0.55	2.79 ± 0.82
Abscess/Blood	0.69 ± 0.22	0.70 ± 0.15	0.58 ± 0.12	0.72 ± 0.13	0.70 ± 0.29

Table S5. Biodistribution results of [^{99m}Tc]Tc-CN5UBI 29-41 in *S. aureus*-infected and turpentine-oil-inflamed mice (mean ± SD, %ID/g, n = 5)

Tissue	*S. aureus*-infected mice model			turpentine-oil-inflamed mice model
	30 min	120 min	240 min	120 min
Heart	1.21 ± 0.14	0.89 ± 0.25	0.58 ± 0.11	0.61 ± 0.03
Liver	2.42 ± 0.14	2.24 ± 0.67	2.23 ± 0.35	1.44 ± 0.23

Tissue	*S. aureus*-infected mice model			turpentine-oil-inflamed mice model
	30 min	120 min	240 min	120 min
Lung	3.09 ± 0.25	1.85 ± 0.29	1.51 ± 0.81	1.26 ± 0.13
Kidney	19.80 ± 2.71	18.00 ± 3.49	17.49 ± 3.81	13.05 ± 1.24
Spleen	1.04 ± 0.30	0.77 ± 0.19	0.74 ± 0.21	0.59 ± 0.10
Stomach	0.43 ± 0.12	0.70 ± 0.24	0.93 ± 0.50	1.82 ± 0.81
Bone	1.67 ± 0.35	1.06 ± 0.14	0.72 ± 0.15	0.82 ± 0.19
Muscle	0.86 ± 0.43	0.34 ± 0.03	0.41 ± 0.06	0.32 ± 0.02
Small Intestines	2.12 ± 0.51	1.65 ± 0.95	0.91 ± 0.39	0.91 ± 0.21
Large Intestines	0.67 ± 0.10	2.23 ± 1.02	2.93 ± 1.50	0.62 ± 0.18
Abscess	1.64 ± 0.40	1.44 ± 0.46	1.42 ± 0.59	0.73 ± 0.05 *
Blood	3.59 ± 0.15	1.81 ± 0.29	1.79 ± 0.64	1.28 ± 0.12
Gallbladder (%ID)	0.03 ± 0.01	0.13 ± 0.10	0.28 ± 0.18	0.15 ± 0.04
Thyroid (%ID)	0.03 ± 0.01	0.02 ± 0.01	0.02 ± 0.00	0.02 ± 0.01
Abscess/Muscle	2.70 ± 0.97	4.15 ± 0.49	3.46 ± 0.67	2.52 ± 0.22 *
Abscess/Blood	0.46 ± 0.12	0.81 ± 0.05	0.79 ± 0.08	0.59 ± 0.06 *

*$P < 0.05$

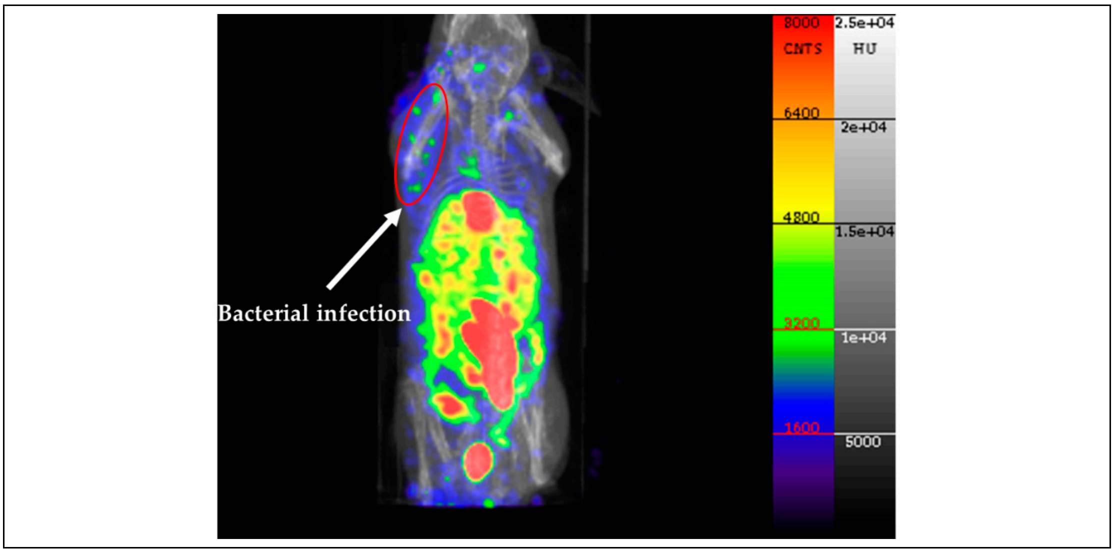

Figure S19. SPECT image of [^{99m}Tc]Tc(CO)$_3$-CN5UBI 29-41 in mice with bacterial infection at 120 min postinjection

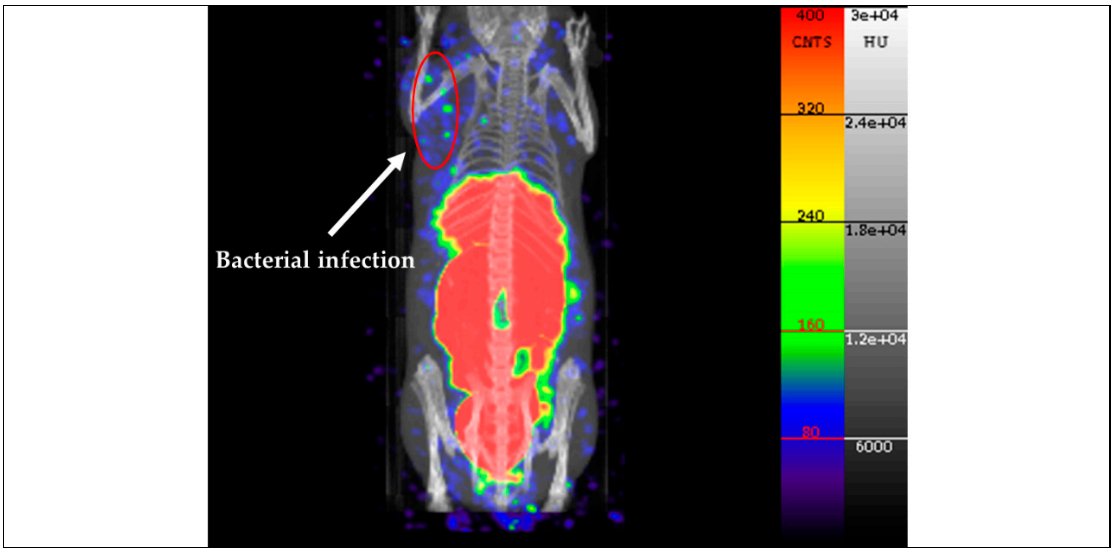

Figure S20. SPECT image of [^{99m}Tc]TcN-CN5UBI 29-41 in mice with bacterial infection at 120 min postinjection
